# Event and Comment

**Published:** 1912-12-15

**Authors:** 


###  DENTAL REGISTER.

**Vol. LXVI. December 15, 1912. No. 12**

### EVENT AND COMMENT.

NATIONAL DENTAL ASSOCIATIONS NEW CONSTITUTION. The constitution adopted by the National Association at its last meeting is too long for us to print, but we shall take space enough to give its outlines, which we trust will be sufficient to give an intelligent conception of the effort made to convert the old Association into an up to date business organization that will attract every professional dentist in the country into its membership. The first and most strenuous contention was over the name, but the old name seemed to be good enough to build the new organization onto. The object stated is to perfect an organization which will unite all Local, District and State society members to membership with the new organization and so make it the largest dental society the world has ever known. All District and State society members if affiliated are eligible, and will be entitled to membership on payment of the annual fee of one dollar, which entitles them to all the privileges of the National and a subscription to the new Journal it is proposed to publish.

A House of Delegates will be created by delegates appointed by the component societies. Each society having 100 members will have two delegates in the House, and one additional for every 200 members above one hundred. This House of Delegates elects from members of the Association and outside of its own members the officers and all the important committeemen and is the court of final appeal in the adjustment of all contentions and business matters of the Association. It has power to change the constitution and By-Laws under certain defined conditions. It is in fact the head of all the executive bodies, which exist and function at its will. It may even create branch organizations if thought expedient. The officers, are a President, three Vice-Presidents, a General Secretary and a Board of nine Trustees, which Board of Trustees elects the Treasurer and other important business committees, and it also cares for the financial and other material interests of the Association. The Trustees are elected outside of the House of Delegates membership, but become ex-officio members with all privilege of the House members, except voting.

The annual dues of one dollar is designed to secure a large membership so as to provide enough funds to enable the Association to print its own Journal. The by-laws cover all the necessary points required to make effective a great organization with complicated functions. The number and diversity of duties makes one feel that all the time of the sessions will be taken up with committee work of some kind.

The connection of the component societies is made close by the fact that when one looses his membership in his state or local society he also looses his membership in the National body. He must also keep his dues paid up promptly or his membership lapses. No one can serve as a delegate who is not a member. Neither can he participate in any of the proceedings. The President presides at all general meetings of the Association and House of Delegates. He can not call special meetings at the request of the House of Delegates, but can at the request of the constituent members. All elections must be by ballot. The President must present an address of not more than 40 minutes at each annual meeting,—this may exclude some great talkers, and workers from the presidency.

The General Secretary is to be the hardest worked man in the Association, but he will be paid a salary if the Trustees can afford it. He writes all the letters and has a general knowledge of all the functions of the organization. The Trustees are nine in number and they have a complete organization of their own with a full set of officers. They appoint the Treasurer and are responsible for all financial and material transactions. They also appoint the editor and business manager of the Journal and hold at least two regular meetings each year to prepare Association programs and business matters.

Three classes of committees are raised by the Trustees: (*a*) Standing Committees; (*b*) Reference Committees; (*c*) Special Committees. The Standing Committees are: 1. Judicial Council; 2 Educational Committee; 3 Legislative Committee; 4 Place of Meeting and Entertainment Committee.

The Judicial Committee of 5 members adjusts all contentions and its acts can only be reversed on appeal to the House of Delegates. The Educational Committee of 5 works to promote creditable educational standards. The Legislative Committee of 5 adjusts all legal affairs and seeks to promote systematic state and national legislation.

The Committee on Place of Meeting is charged with the duty of making all arrangements for the annual sessions of the Association, except the programme making.

The Reference Committe has general charge of the Section work; provides rules of order for conduct of the business of the sessions; general oversight of educational affairs; legislative affairs; amendment to constitution and by-laws; official reports; credentials and miscellaneous business.

A general meeting will be held the first day at 10:30 a. m. and a paper will be presented and discussed each evening selected by one of the three Sections. All other papers will be read before the Sections, and the rest of the time will be utilized for giving clinics, etc.

Each of the three Sections will be organized with a chairman, vice-chairman, secretary and executive committee. All papers or abstracts of papers to be read before the Association whether read or ordered printed must be in the hands of the secretary of the Section, where they are read, at least 35 days before the meeting. Papers will be limited in length of reading to 35 minutes, and only five minutes will be given to each discussor. All papers must be approved by the Section officers before they are put on the programme, or read and published. No paper will be published and credited to the Association which has not been read before it. Any author may have the privilege of having his paper published in other than the Association Journal.

All resolutions must be approved by the House of Delegates before they can be printed in the name of the National Dental Association.

The by-laws may be amended at any session daily if desired by a two-third vote of the House of Delegates.

This seems like a lot of machinery, but it is a full grown job we are undertaking and will need all our wisest plans and facilities. It ought to be successful if everybody cooperates willingly and faithfully.

OHIO STATE MEETING. The experiment of holding the meeting of the Ohio State Dental Society in different places each year was inaugurated most successfully in the meeting just held at Cincinnati, if success can be predicted and justified on the basis usually reckoned with. The attendance was probably the largest in the Society's history. It was estimated that 1500 dentists were in attendance. They came from all parts of the State, and the adjoining States of Kentucky, West Virginia, and Indiana sent a large number of visitors.

The program of papers, lectures and clinics was of unusual merit and doubtless had much to do with bringing so many dentists together. The exhibits by dealers and manufacturers were very attractive, we have seldom seen a larger and greater variety of exhibits anywhere, and those who value such things could not help being satisfied. We could not notice a single line that was not well represented. The place of holding the meeting was ideal. The whole ninth floor of the great Sinton Hotel was given over to the meeting.

The large hall accommodated the meeting perfectly and served as an excellent room for the clinics. It had the advantage of being free from street noises which usually make it almost impossible to hear what is being said. The long corridors with their exhibit rooms connected made an ideal arrangement, as it was possible for those who did not care to attend the sessions to spend their time with the exhibitors without disturbing the sessions. The meeting next year will go to Toledo and the year after to Columbus, as it is planned to have the Miller Memorial monument ready for dedication at that time.

The State Society has so far perfected its reorganization that it now boasts of a membership of over nine hundred. and decided by a unanimous vote to enter the National Dental Association as a body. It is the first State Society to take this step and if a few more of the strong State Societies take a similar action the success of the National reorganization will be assured. This will undoubtedly occur as rapidly as other State Societies hold their annual meetings, as the recent action of the National makes this easy. We havn't space here to even mention all the good things done in the way of papers, clinics and lectures; but we were especially entertained and instructed by the moving picture lecture as presented by the Oral Hygiene Committee and the lecture on the "venereal peril" as shown in the pictured lecture by a representative of the National Cash Register Co., of Dayton, Ohio. It was a most remarkable production and ought to be widely exhibited. The exhibit of the work being done by the Cincinnati Odontological Society in the public schools of Cincinnati was also extremely interesting and Dr. Sidney Raue and other dentists working with him deserve much credit for the admirable and sensible way in which they are undertaking this problem.

The following officers were elected for next year: President, W. A. Price; First Vice-President, J. K. Douglas; Second Vice-President, E. C. Mills; Directors, W. H. Whitslar, L. L. Barber, H. E. Biermann, H. C. Brown. Toledo will have to work to equal the Cincinnati meeting.

UNIFORMITY IN STATE BOARD EXAMINATIONS. The President of the National Association of Dental Examiners at the recent annual meeting urged a plan of making more stringent and uniform registration requirements. Just how this can be accomplished, in view of the fact that State Examining Boards are continually changing in personel, and there is no national legislation to compel concerted action, seems to make such a plan improbable, although it would be highly desirable, not only in the interest of higher standards, but also to facilitate reciprocal changes. It is very desirable that some means shall be found to accomplish this object.

UNIFORMITY OF PROFESSIONAL LAWS. The Dental Educational Council, at its recent meeting in Washington got the Faculties Association and Examiner Association to join the National Dental Association in an effort to devise a uniform dental bill to be submitted to the several states to serve as a model for each state, so that the standard of registration in each state will be as nearly like that of all other states as to make reciprocity or exchange of licensed dentists more practicable without examinations. It is proposed to enlist the cooperation also of the American Bar Association. This certainly is a move in the right direction and would seem that there ought not to be any great difficulty in framing a satisfactory bill that would establish a general minimum of standard that would serve for all states. The administration of the bill could be made more exacting in such states as could stand for a higher standard. This would give us a national standard.

